# Environmentally Friendly Water-Based Reduced Graphene Oxide/Cellulose Nanofiber Ink for Supercapacitor Electrode Applications

**DOI:** 10.1021/acsomega.3c09139

**Published:** 2024-02-27

**Authors:** Kiran I. Nargatti, Sandeep S. Ahankari, John Ryan C. Dizon, Ramesh T. Subramaniam

**Affiliations:** † School of Mechanical Engineering, Vellore Institute of Technology, Vellore, Tamil Nadu 632014, India; ‡ DR3AM Center, Bataan Peninsula State University-Main Campus, City of Balanga, Bataan 2100, Philippines; § Department of Physics, Faculty of Science, Universiti Malaya, Kuala Lumpur 50603, Malaysia

## Abstract

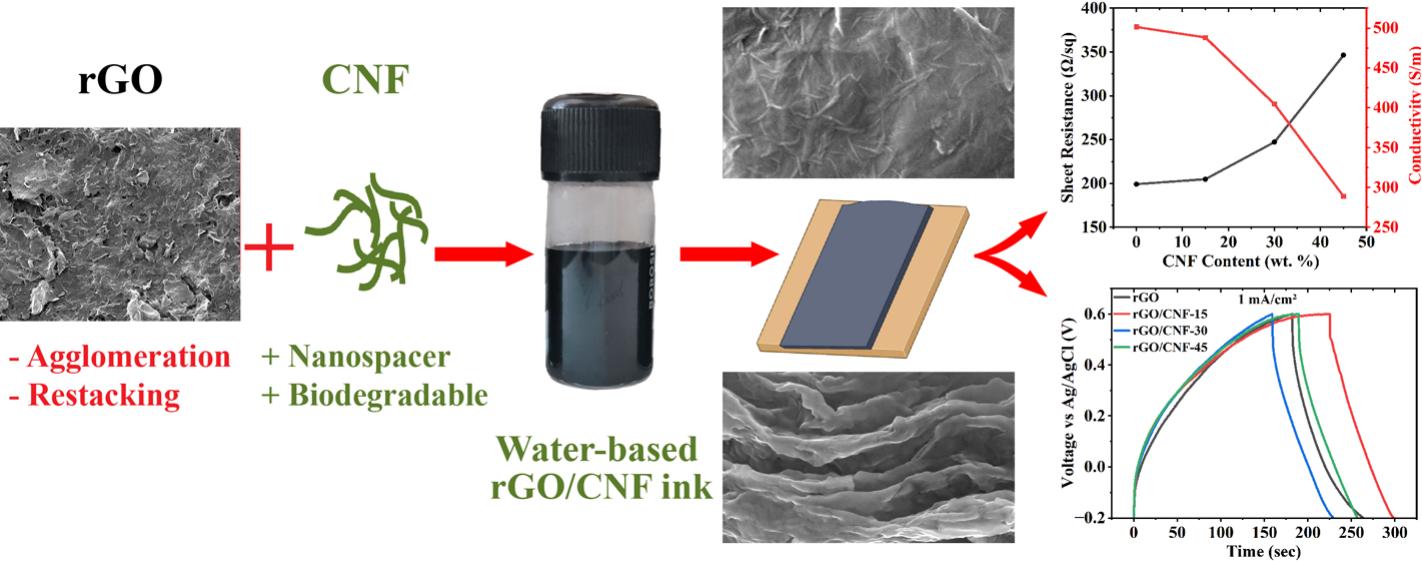

The agglomeration of reduced graphene oxide (rGO) in water makes the development of rGO inks for supercapacitor printing challenging. Cellulose nanofiber (CNF), a biodegradable and renewable nanomaterial, can act as a nanospacer, preventing the agglomeration and restacking of rGO flakes. In this work, rGO/CNF films were fabricated using an environmentally friendly water-based rGO/CNF ink. In the absence of an additional binder/surfactant, the rGO/CNF films demonstrated remarkably enhanced hydrophilicity while retaining good electrical conductivity. The concentration of CNF was varied to observe the variation in the electrochemical performance. At a current density of 1 mA/cm^2^, the rGO/CNF-15 film exhibited a maximum areal capacitance of 98.61 mF/cm^2^, closely matching that of pure rGO films. Because of its excellent electrical performance, ease of manufacturing, and environmental friendliness, this water-based rGO/CNF ink may have promising applications in the printing of supercapacitor electrodes.

## 1 Introduction

In recent years, there has been growing interest in and demand for printed miniature electronic devices that use high-performance materials. As a result, extensive research has been conducted on carbonaceous materials (such as graphene derivatives, activated carbon, and carbon black) and other 2D-layered material-based inks. The unique physicochemical properties and thickness-dependent electrical and optical properties of these 2D nanomaterials have expanded the possibilities of utilization of these 2D nanomaterials in the field of printed miniature electronic devices.^1^ Graphene and its derivatives, graphene oxide (GO) and reduced graphene oxide (rGO), are one-atom thick sheets of carbon atoms arranged in a hexagonal lattice. rGO is preferred for printed electronic devices in the field of energy storage/generation,^2,3^ electromagnetic interference shielding,^4^ sensors,^5^ and bioelectronics^6^ due to their large surface area (∼500 m^2^/g) and good electrical and thermal conductivity. However, rGO flakes tend to agglomerate due to the strong van der Waals forces and π–π stacking interaction. This agglomeration of rGO flakes is an immediate issue that needs to be addressed as it reduces the surface area, makes it harder for electrical current to pass through them, and also prevents ion diffusion in the case of energy storage devices such as supercapacitors.^7,8^ One effective way to solve this issue is to introduce electrochemically active compounds, such as carbon nanotubes,^9^ carbon black,^10^ redox-active nickel ferricyanide,^11^ conducting polymers, e.g., polyaniline^12^ and polypyrrole,^13^ and transition-metal oxides, e.g., MoO_3_,^14^ TiO_2_,^15^ as a nanospacer between the rGO flakes for preventing their restacking and improving the performance of the devices.

Another challenge with rGO is the solvent used for the development of the rGO inks. The most commonly used solvents for rGO dispersion are organic solvents such as dimethylformamide, *N*-methyl-2-pyrrolidone, glycol, ethanol, and dimethyl sulfoxide.^16^ However, most of them are volatile and toxic, have a high boiling point, and are harmful to the environment. Therefore, there is a need to replace them with more environmentally friendly solvents. Water can be an ideal solvent since it is nontoxic, has an optimum boiling point, and can replace organic solvents. However, rGO is hydrophobic, which makes it difficult to disperse in water.^17^

Considering the need for environmentally friendly materials and processes, one effective approach to address the aforementioned challenges is using cellulose-derived materials like cellulose nanofibers (CNFs).^18,19^ CNF has gained interest as a sustainable nanomaterial due to its biodegradability, biocompatibility, and natural abundance. The unique structure and properties of CNF, such as its high aspect ratio with diameter <100 nm, large surface area, and strong hydrophilicity due to the presence of abundant hydroxyl groups on the surface can help to strongly interact with water.^20^ Additionally, CNF can act as nanospacers between rGO flakes, allowing graphene nanoparticles to be stabilized in water.^21^

In this study, rGO/CNF film electrodes were fabricated using water-based ink with different compositions of rGO and CNF. These electrodes were developed using the doctor blade technique. The morphological and electrochemical properties of the developed ink and film electrodes were carried out for all rGO/CNF ratios. The main focus of the work was to study the effect of the CNF concentration on the microstructure, electrical conductivity, and electrochemical performance of the water-based rGO/CNF composite films. The water-based rGO/CNF film with optimal CNF content can facilitate efficient electron transport throughout the material and increase hydrophilicity while maintaining the electrical conductivity of the film. This ink can be utilized to produce flexible and scalable electrodes with adjustable morphology and electrochemical performance using a variety of printing methods, including screen, spray, and inkjet printing.^22^ As the ink uses water as its main solvent instead of hazardous organic solvents, it is a more environmentally friendly and sustainable option. Consequently, this novel ink paves the way for the development of high-performance, environmentally friendly, and cost-effective supercapacitors with significant potential to transform energy storage in various applications, such as wearable electronics and health monitoring devices.

## 2 Experimental Section

### 2.1 Materials and Chemicals

rGO was obtained from Carborundum Universal Ltd., India and has the following specifications: less than 5 layers, bulk density ∼0.001 g/cm^3^, surface area ∼500–600 m^2^/g. rGO was directly used without any further treatment. CNF (purity >99.9%) was purchased from Intelligent Materials Pvt. Ltd. (Nanoshel), India. Deionized (DI) water (Milli-Q, Millipore) was used for the preparation of the solution.

### 2.2 Preparation of the rGO/CNF Films

**Figure 1:**
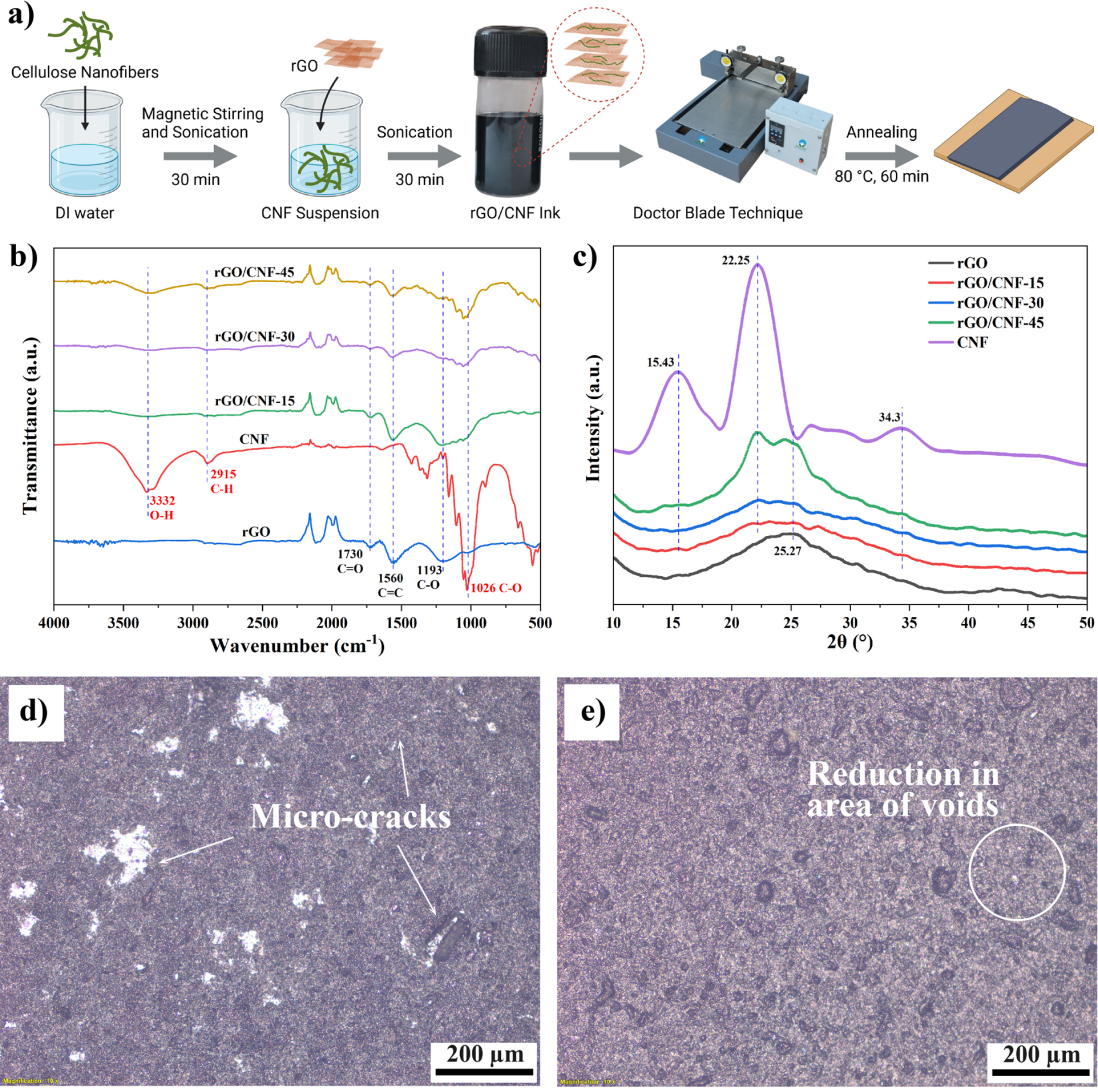
(a) Schematic illustration of rGO/CNF ink formulation and fabrication of rGO/CNF film electrodes, (b) FTIR spectra, and (c) XRD of rGO, rGO/CNF-15, rGO/CNF-30, and rGO/CNF-45 films. Optical images of (d) rGO and (e) rGO/CNF-45 films.

Figure 1a schematically illustrates the formulation of the rGO/CNF ink and the fabrication of rGO/CNF films. To develop different rGO/CNF inks with rGO to CNF weight ratios of 85:15, 70:30, and 55:45, different CNF solutions were first prepared by dispersing their appropriate amounts in 20 mL of DI water using a magnetic stirrer for 20 min at room temperature. To achieve uniform CNF dispersion, solutions were kept under an ultrasonic probe sonicator (Johnson Plastosonic ULP 500 model, operated at 60% of its maximum amplitude with 5 s ON and 5 s OFF intervals) at 20 kHz for 30 min. To prepare the rGO/CNF inks, 0.2 g of the as-received rGO was mixed into the CNF solutions, and the mixtures were left under a probe sonicator for 30 min to achieve nanofiller dispersion and deagglomeration of rGO. Films were developed using the doctor blade technique [Flatsheet Membrane Casting machine, Tech Inc., Chennai, India (height control −10 μm)], followed by annealing in an oven at 80 °C for 60 min. The films were named as rGO/CNF-15, rGO/CNF-30, and rGO/CNF-45. For comparison, the rGO film was also prepared similarly to the above method.

### 2.3 Characterization

#### 2.3.1 Morphological Characterization

Functional groups of the rGO/CNF inks were analyzed using Fourier transform infrared (FTIR) spectroscopy (IRAffinity-1, Shimadzu, Japan). The X-ray diffraction (XRD) spectra of the rGO/CNF films were measured using an X-ray diffractometer (PANalytical- X’Pert^3^ Powder) with reference target: Cu Kα radiation; voltage, 35 kV; current, 30 mA. Morphological study of the deposited films was conducted by using both optical and field emission scanning electron microscopy (SEM). Optical images were taken with a KEYENCE, VHX-7000, and ZEISS OLYMPUS BX53M. SEM images were taken using FEI Quanta 250 FEG model working in the secondary electron mode at an accelerating voltage of 20 kV. Hydrophilicity of the films was analyzed by water contact angle using the sessile drop method (Kruss-DSA25).

#### 2.3.2 Electrical and Electrochemical Characterization

The sheet resistance of the films was measured using a four-point probe setup (model DFP-02, SES) with a probe diameter of 0.9 mm and a probe spacing of 2 mm. The voltage was measured while the electrical current was passed through the films. The sheet resistance (ρ_s_) in Ω/sq was calculated using eq 1, and the electrical conductivity (σ) of the films in Ω/m was obtained using eq 2.
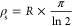
1
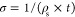
2Here, *R* is the film resistance calculated from the ratio of *V*/*I*.  is the geometrical correction factor equal to 4.532 considered based on geometry and dimensions of films, and *t* is the film thickness 10 ± 4 μm (Figure S1).^23,24^ The electrochemical performances of the rGO/CNF films were studied using a Biologic SP-200 potentiostat. The electrochemical properties of the films were analyzed with a standard three-electrode cell using Pt foil as a counter electrode, Ag/AgCl (3 M KCl) as a reference electrode, and 1 M KOH aqueous solution as an electrolyte. Cyclic voltammetry (CV) analysis was carried out in the potential range from −0.2 to 0.6 V at different scan rates of 5, 10, 20, 30, and 50 mV/s. Galvanostatic charge–discharge (GCD) analysis was carried out in the potential range of −0.2 to 0.6 V at different current densities of 1, 2.5, 5, and 10 mA/cm^2^. Electrochemical impedance spectroscopy (EIS) was carried out over a frequency range from 0.1 Hz to 1 MHz at a voltage amplitude of 10 mV. The areal capacitance *C*_A_ (mF/cm^2^) is calculated from the GCD curves according to the following equation.^25,26^
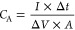
3where *I* (mA) is the discharge current, Δ*t* (sec) is the discharge time, Δ*V* (V) is the potential window, and *A* (cm^2^) is the area of the working electrode.

## 3 Results and Discussion

### 3.1 Morphological Characterization

The FTIR spectra of the CNF powder, rGO, rGO/CNF-15, rGO/CNF-30, and rGO/CNF-45 films are displayed in Figure 1b. The FTIR spectra of CNF show characteristic peaks at 3332, 2915, and 1026 cm^–1^. These peaks correspond to typical stretching vibrations of hydroxyl groups (−OH), aliphatic C–H stretching, and C–O stretching vibrations, respectively. For rGO films, peaks at 1730, 1560, and 1193 cm^–1^ correspond to the stretching vibrations of C=O, C=C, and C–O,^13,27^ respectively. For the rGO/CNF-15, rGO/CNF-30, and rGO/CNF-45 films, the intensity of characteristic peaks at 3332 and 2915 cm^–1^ was found to increase significantly with increasing CNF concentration. Whereas, the characteristic peaks of rGO corresponding to C=O, C=C, and C–O stretching decreased significantly. This was attributed to the presence of the CNF at the rGO surface, which led to the detection of the CNF functional group despite the formation of the nanocomposite. Notably, the intensity of characteristic CNF peaks at around 3332 (O–H), 2915 (C–H), and 1026 cm^–1^ (C–O) significantly weakens in the FTIR spectrum of rGO/CNF-45. This could be due to CNF intercalating between rGO sheets, which disrupts the ordered structure of CNF and reduces its crystallinity, resulting in broader and weaker peaks. Another possible reason is the formation of hydrogen bonds between the hydroxyl groups of CNF and the oxygen-containing functional groups of rGO, such as epoxy and carboxyl groups. This lowers the intensity of the peaks corresponding to these groups and shifts their positions slightly. Figure 1c shows the XRD patterns of the rGO, CNF, rGO/CNF-15, rGO/CNF-30, and rGO/CNF-45 films. The diffraction peaks of CNF at 15.43, 22.25, and 34.3° correspond to the (101), (200), and (004) diffraction planes of the cellulose I crystal structure, respectively.^28,29^ The wider peak at 25.27° demonstrates the obvious characteristics of rGO, which correspond to the (002) plane of graphite and indicate the reduction of GO. The XRD spectra of the rGO/CNF composites exhibit additional peaks at an approximate angle of 22°, which can be attributed to the incorporation of CNF within the composite material.^27^ With increasing CNF content, the diffraction peak of rGO gradually shifts to the left, and the intensity of CNF’s diffraction peaks increases, indicating that CNFs are well preserved in the rGO/CNF composites.^30,31^ The XRD results confirm the observations made with FTIR.

The surface morphology of these rGO/CNF films was analyzed by both optical and SEM images. As confirmed by optical images, the rGO film (see Figure 1d) shows the formation of microcracks in the film, which probably formed due to the high degree of agglomeration of rGO. The incorporation of CNF has been found to have a positive impact on the dispersion of rGO flakes in the film, as shown in Figure 1e. One can see the reduction in the size and area of the voids; which can be attributed to the reduction of rGO agglomeration and facilitated the formation of a uniform film (Figure S1). To analyze the morphology in detail, SEM analysis has been carried out.

**Figure 2:**
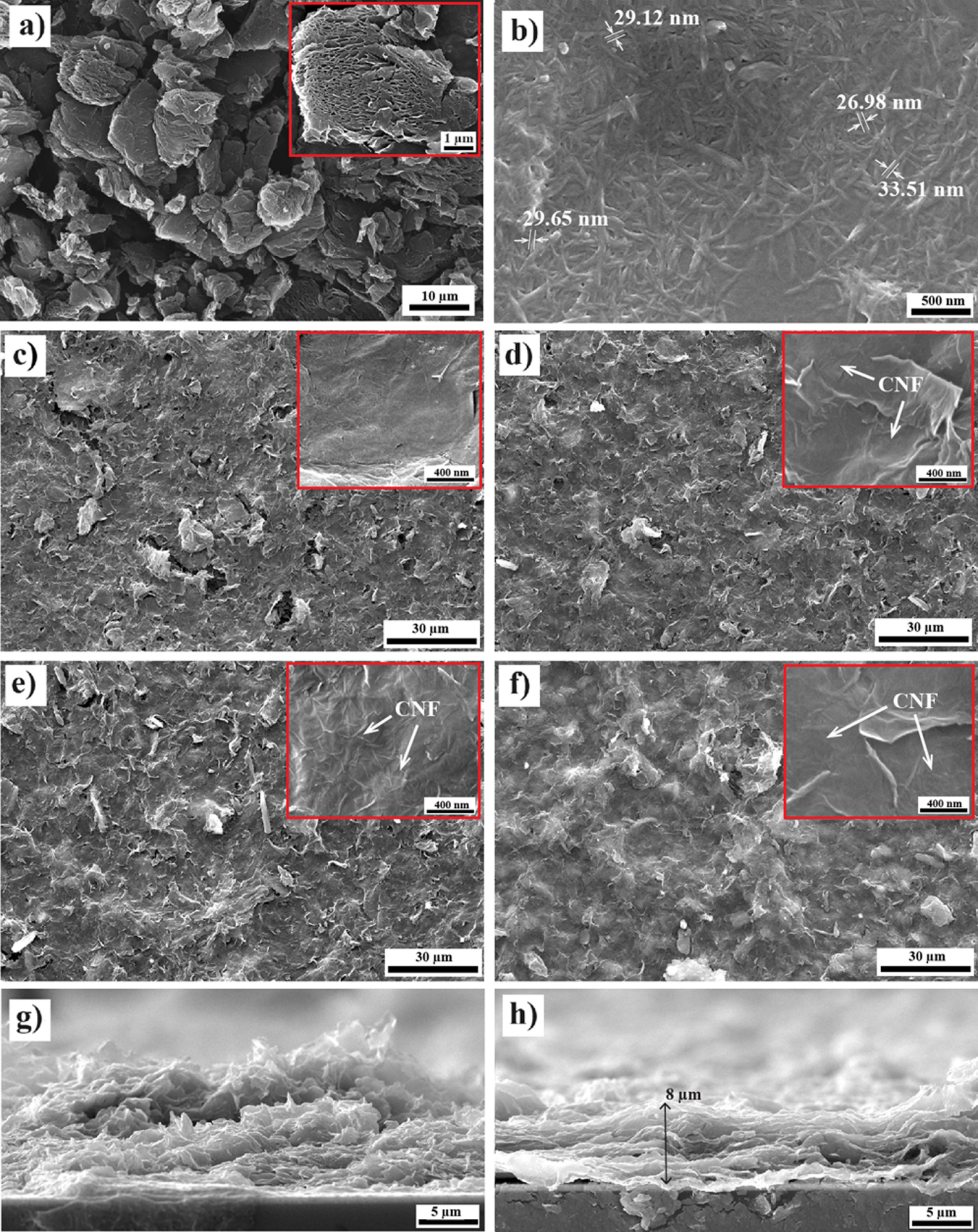
SEM images of as-received (a) rGO powder, (b) CNF; (c) rGO, (d) rGO/CNF-15, (e) rGO/CNF-30, and (f) rGO/CNF-45 films; cross section of (g) rGO and (h) rGO/CNF-30 films.

The SEM image of the as-received rGO powder shows agglomerated flakes that are tightly packed and appear to be stacked on top of each other, as shown in Figure 2a. The lateral size of the rGO flakes is approximately 1–3 μm. Figure 2b shows the SEM image of the CNF with a diameter of nearly 30 nm and a length of several microns. These nanofibers are randomly oriented and appear to be well dispersed. Figure 2c shows the SEM image of rGO film, which confirms the wrinkled and folded characteristic of rGO.^32^ It can be clearly seen that the rGO films have cracks and voids that are attributed to the van der Waals force and interplanar π–π interaction between rGO flakes. Additionally, the cross-section of the rGO film (Figure 2g) clearly shows the disordered and stacked rGO flakes. This can reduce the electrical conductivity of the film, as it does not form a connecting pathway for the flow of electrons. The SEM image of rGO/CNF films shows uniformly dispersed rGO and CNF randomly covering the rGO surface, as shown in Figure 2d–f. The addition of CNFs effectively prevents the restacking of the rGO flakes and weakens graphene interplanar π–π interaction, resulting in ordered stacked rGO flakes in the rGO/CNF films, as shown in Figure 2h.^33^ This significantly increases the contact area between the layered RGO flakes, reducing the barrier for electron transfer and improving the electrical conductivity of the film.^12,34,35^ This will assist in resolving the issue of agglomeration between graphene flake layers and a specific area significantly below the theoretical value. As the CNF content in the rGO/CNF composite increased, a corresponding increase in the density of CNF on the rGO surface was observed. SEM analysis of the rGO/CNF-15 composite, shown in Figure 2d, revealed a low density of CNF lightly distributed on rGO flakes. In contrast, Figure 2e illustrates the rGO/CNF-30 film displaying a uniform dispersion of CNF on the rGO flakes. However, with a higher CNF content in the rGO/CNF-45 film, as shown in Figure 2f, the density of CNF increased significantly, leading to its noticeable agglomeration, which led to a detrimental effect on electrical conductivity. SEM results corroborated the observations made by optical microscopy.

**Figure 3:**
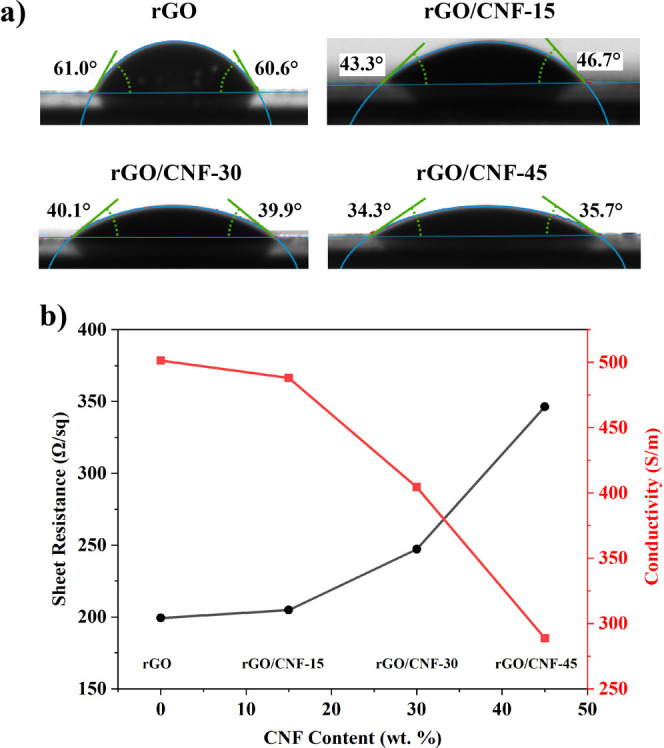
(a) Water contact angle and (b) sheet resistance and electrical conductivity of rGO, rGO/CNF-15, rGO/CNF-30, and rGO/CNF-45 films.

The water contact angle is a measure of the surface hydrophilicity, which influences the electrolyte wettability and adsorption to the electrode material. As shown in Figure 3a, the water contact angle of pure rGO film was 61°, whereas the addition of CNF in water-based rGO ink resulted in a significant reduction in the contact angles to 46.7, 40.1, and 35.7° for rGO/CNF-15, rGO/CNF-30, and rGO/CNF-45 films, respectively. This notable decrease in the contact angle indicated a substantial increase in the hydrophilicity of the films. This can be attributed to the abundant hydroxyl groups on the surface of the CNF, which can form hydrogen bonds with water molecules and increase the polarity of the rGO/CNF composite film. This increased hydrophilicity promotes the electrolyte wettability and, consequently, facilitates ion transport, thus enhancing the charge storage ability of the electrode.

### 3.2 Electrical and Electrochemical Characterization

Figure 3b correlates the CNF concentration with the electrical properties of the rGO/CNF films. As the CNF content increases, the sheet resistance of the film increases, and the electrical conductivity decreases. The electrical conductivity marginally decreased from 501.44 S/m for pure rGO film to 488.11 S/m for the rGO/CNF-15 film and further to 404.5 S/m for the rGO/CNF-30 film. However, the introduction of a higher CNF content in the rGO/CNF-45 film resulted in a significant drop in electrical conductivity, down to 288.73 S/m, attributed to the high electrical insulation of CNF which hinders the flow of charge carriers.^27^ It is noteworthy that there is a trade-off between hydrophilicity and electrical conductivity in these films. These results suggest that the CNF content should be carefully optimized while striking a balance between hydrophilicity and maintaining adequate electrical conductivity for optimal supercapacitor performance.

**Figure 4:**
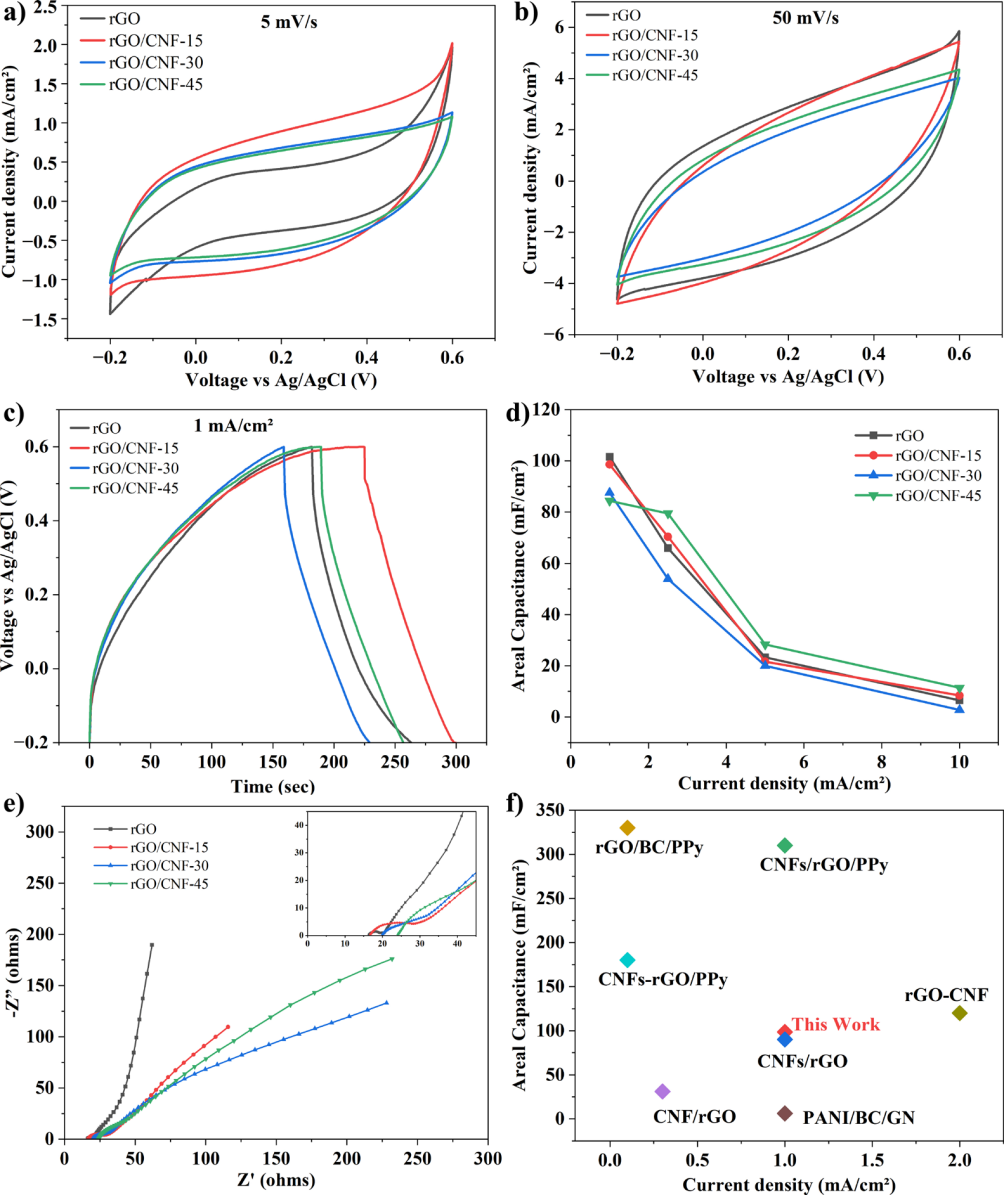
CV curves at scan rates of (a) 5 and (b) 50 mV/s, (c) GCD curve at current density of 1 mA/cm^2^, (d) areal capacitance as a function of the current density, (e) Nyquist plot for different water-based rGO/CNF film electrodes, and (f) comparison of areal capacitance of different rGO/CNF-based supercapacitor electrodes.^13,27,38−42^

The electrochemical performance of all prepared electrode films was characterized via CV and GCD analysis. Figure 4a,b shows the CV curves of rGO, rGO/CNF-15, rGO/CNF-30, and rGO/CNF-45 film electrodes at scan rates of 5 and 50 mV/s. As per the CV curves, all of the samples at 5 mV/s are virtually rectangular as well as nearly symmetric, indicating the perfect supercapacitive performance of the electrodes. Compared with the CV curve of the rGO film, the CV curve of the rGO/CNF-15 film exhibited a larger rectangular area. This is attributed to the facilitation of uniform film formation with the addition of CNF as a nanospacer, consequently increasing the specific surface area and hydrophilicity while maintaining the electrical conductivity of the rGO film. The increased surface area provides more active sites for electrochemical reactions, while the higher electrical conductivity improves charge transport within the film. As a result, the rGO/CNF-15 film exhibits a higher areal capacitance than the rGO film. This makes it a promising material for use in supercapacitors and other energy storage devices. However, with increasing CNF content further, the area of the CV curve becomes smaller. The CV curves of all samples at different scan rates are shown in Figure S2. It can be seen that, with an increased scanning rate, the electrode’s current density was similar to that observed for typical electric double-layer capacitors (EDLCs).^36^ At higher scan rate, the CV curve altered slowly to a quasi-rectangular shape because of the insufficient diffusion of the ions of the electrolyte.^37^

GCD performance of the water-based film electrode was measured to analyze the capacitive performance of the electrodes. The GCD curves at 1 mA/cm^2^ current density (see Figure 4c) are roughly triangular and symmetrical. The charge/discharge time of rGO/CNF-15 films is found to be longer than that of pure rGO and other rGO/CNF films, indicating a higher areal capacitance. The GCD curves of all samples at different current densities are shown in Figure S3. The voltage drop (*iR*) at the beginning of the discharge curve due to internal resistance was 0.05, 0.06, 0.13, and 0.12 V for the rGO, rGO/CNF-15, rGO/CNF-30, and rGO/CNF-45 film electrodes, respectively. The higher conductivity of the pure rGO film contributed to a smaller *iR* drop, whereas the lowest conductivity rGO/CNF-45 film displayed a significantly higher *iR* drop. This confirmed the expected correlation between electrical conductivity and *iR* drop. The areal capacitance of the film electrodes at various current densities was calculated using 3 and the values are shown in Figure 4d. At 1 mA/cm^2^, rGO, rGO/CNF-15, rGO/CNF-30, and rGO/CNF-45 film electrodes exhibited areal capacitance of 101.54, 98.61, 87.54, and 84.43 mF/cm^2^, respectively. The areal capacitance values match well with the electrical conductivity and CV curve results. The rGO/CNF-15 exhibited nearly similar performance to that of rGO. However, with increasing CNF content, the capacitance decreased. The possible reason for this is the excessive CNF content that reduces the electrical conductivity of the films, limiting the charge transfer and ion diffusion in the electrodes. Even though the capacitance is decreasing with CNF content, this performance of the water-based rGO/CNF films is noteworthy in the absence of any conductive material or binder/surfactant (see Figure 4f).^12,13,27,38−42^

The equivalent-series resistance (*R*_ESR_) and ion-transfer behavior of the rGO/CNF films were examined using EIS. Figure 4e shows the Nyquist plot of the electrodes recorded over the frequency range of 0.1 Hz to 1 MHz. The *R*_ESR_ values of the rGO, rGO/CNF-15, rGO/CNF-30, and rGO/CNF-45 film electrodes, obtained from the intercepts on the real axis, were 16.31, 16.72, 19.92, and 24.15 Ω, respectively. The diameter of the semicircle in the high-frequency region represents the charge transfer resistance (*R*_ct_) and the straight line in the low-frequency region is attributed to Warburg impedance, related to ion diffusion in the electrolyte. The zoomed-in view of the semicircle region reveals that the rGO electrode had the smallest and nearly semicircle, indicating the lowest *R*_ct_ and most ideal capacitive behavior. In contrast, rGO/CNF electrodes had a slightly larger and more distorted semicircle, indicating a higher *R*_ct_, slower charge transfer, and lower capacitive performance. The rGO/CNF electrodes also had steeper slopes in the low-frequency region, suggesting greater Warburg impedance and hindered ion diffusion. The increase in *R*_ESR_ and *R*_ct_ with increasing CNF content was consistent with a decrease in electrical conductivity and capacitance values of the rGO/CNF electrodes. This suggests that incorporating CNFs may impede electron transport to some extent. It is important to optimize the CNF concentration in order to maximize the benefits of the CNFs while minimizing their negative effects. Based on the electrochemical characterization results, 10–15% CNF concentration would be optimal for achieving a balance between the hydrophilicity and electrochemical performance of the films.

## 4 Conclusions

Environmentally friendly water-based rGO/CNF conductive inks with different CNF concentrations were successfully employed to fabricate supercapacitor electrode films by using the doctor blade technique. The addition of green CNF effectively prevents the agglomeration of rGO flakes and the formation of microcracks and voids through the film. The rGO, rGO/CNF-15, rGO/CNF-30, and rGO/CNF-45 films exhibited electrical conductivities of 501.4, 488.1, 404.5, and 288.7 S/m, respectively. Notably, rGO/CNF-15 achieved near-identical electrochemical performance to pure rGO with maximum areal capacitance of 98.61 mF/cm^2^ at a current density of 1 mA/cm^2^. This study highlights the significance of optimizing the CNF content (∼15%) in rGO ink to balance the hydrophilicity and electrical conductivity of the resultant rGO/CNF films for the fabrication of high-performance, environmentally friendly, and cost-effective supercapacitor electrodes.

## Supporting Information Available

Optical images of rGO/CNF-15 and rGO/CNF-30 films and roughness profile of the rGO, rGO/CNF-15, rGO/CNF-30, and rGO/CNF-45 films; CV curves of rGO, rGO/CNF-15, rGO/CNF-30, and rGO/CNF-45 film electrodes at different scan rates from 5 to 50 mV/s; and GCD curves of rGO, rGO/CNF-15, rGO/CNF-30, and rGO/CNF-45 film electrodes at different current densities from 1 to 10 mA/cm^2^ (PDF)
