# Supplementary material for: Environmentally Friendly Water-Based Reduced Graphene Oxide/Cellulose Nanofiber Ink for Supercapacitor Electrode Applications
Source: ACS Omega. 2024 Feb 27;9(10):11730–7. doi: 10.1021/acsomega.3c09139 (PMC10938331; doi:10.1021/acsomega.3c09139)
Supplement: Supplementary file 1 — ao3c09139_si_001.pdf [file ao3c09139_si_001.pdf]

Supporting Information

# Environmentally Friendly Water-Based Reduced Graphene Oxide/ Cellulose Nanofiber Ink for Supercapacitor Electrode Applications

*Kiran I. Nargatti* [a], *Sandeep S. Ahankari* [a,*], *John Ryan C. Dizon* [b], *Ramesh T. Subramaniam* [c]

[a] School of Mechanical Engineering, Vellore Institute of Technology, Vellore, Tamil Nadu, 632014, India.

[b] DR3AM Center, Bataan Peninsula State University-Main Campus, 2100, City of Balanga, Bataan, Philippines.

[c] Department of Physics, Faculty of Science, Universiti Malaya, 50603 Kuala Lumpur, Malaysia.

**Corresponding Author -** asandeep.s@vit.ac.in (Dr. Sandeep Ahankari)

**Table of illustrations:**

**Figure S1.** Optical images of a) rGO/CNF-15 and b) rGO/CNF-30 films, Roughness profile of the c) rGO, d) rGO/CNF-15, e) rGO/CNF-30, and f) rGO/CNF-45 films

**Figure S2.** Cyclic voltammetry (CV) curves of a) rGO, b) rGO/CNF-15, c) rGO/CNF-30, and d) rGO/CNF-45 film electrodes at different scan rates from 5 to 50 mV/s.

**Figure S3.** GCD curves of a) rGO, b) rGO/CNF-15, c) rGO/CNF-30, and d) rGO/CNF-45 film electrodes at different current densities from 1 to 10 mA/cm$^2$.

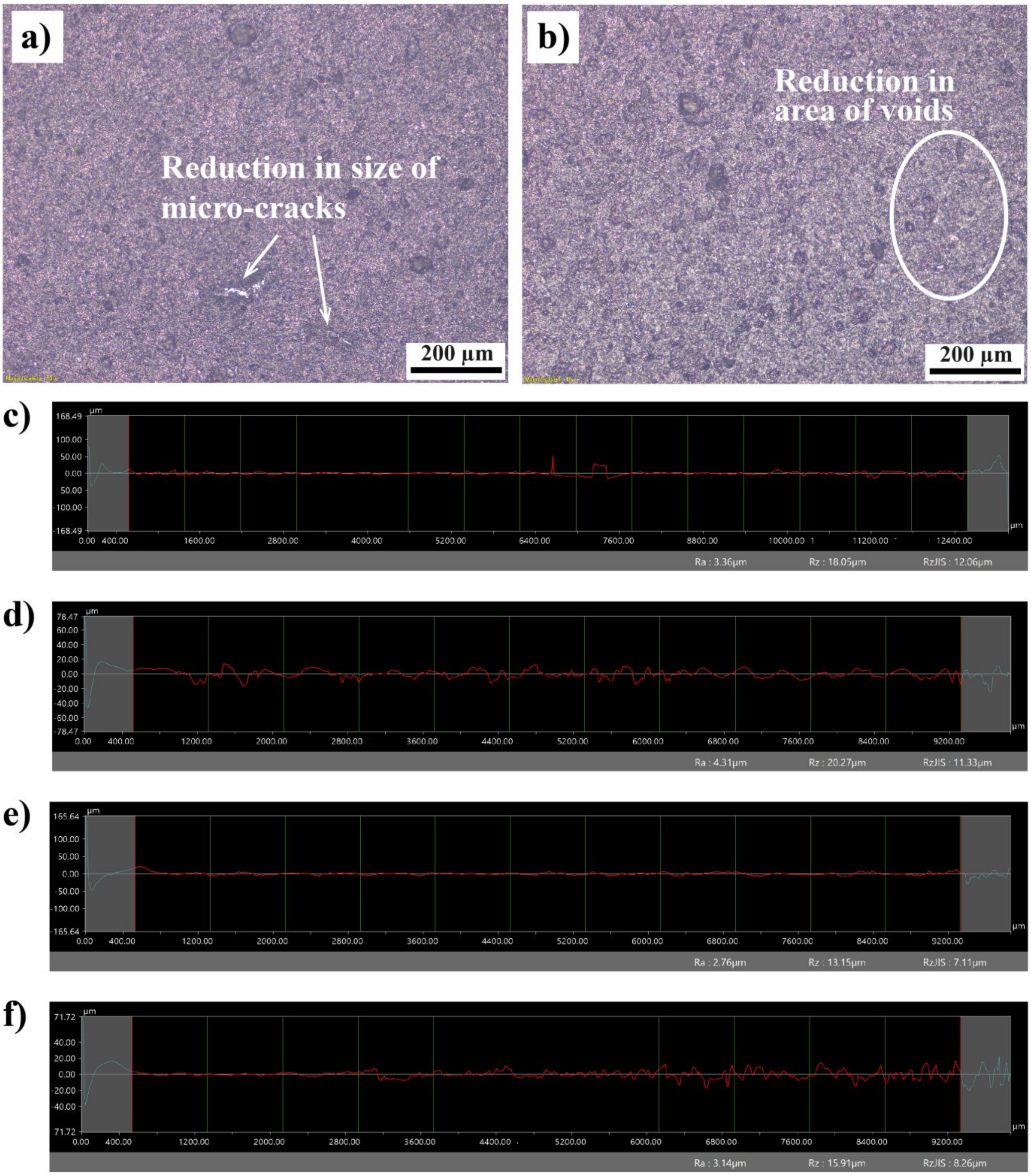


**Figure S1.** Optical images of a) rGO/CNF-15 and b) rGO/CNF-30 films, Roughness profile of the c) rGO, d) rGO/CNF-15, e) rGO/CNF-30, and f) rGO/CNF-45 films

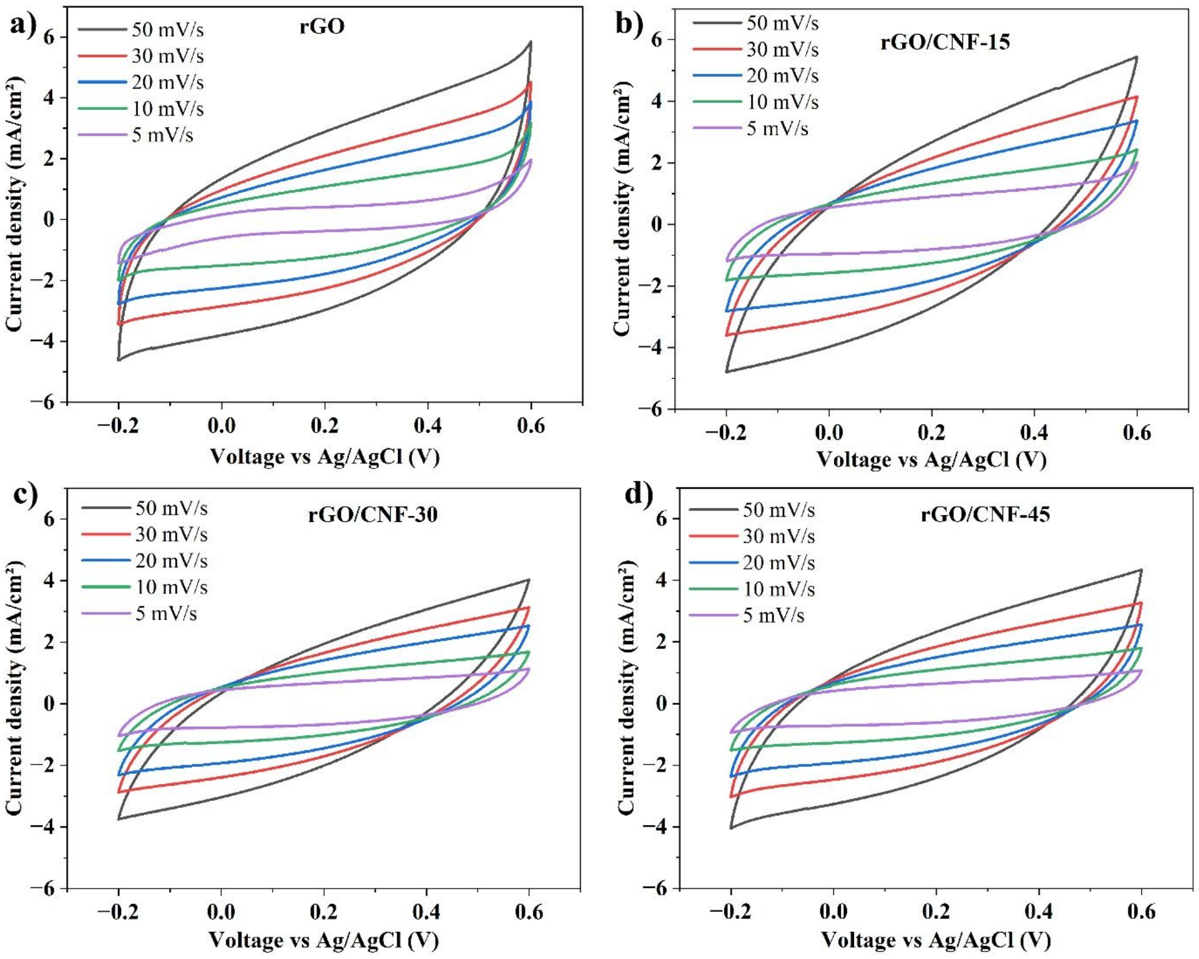


**Figure S2.** Cyclic voltammetry (CV) curves of a) rGO, b) rGO/CNF-15, c) rGO/CNF-30, and d) rGO/CNF-45 film electrodes at different scan rates from 5 to 50 mV/s.

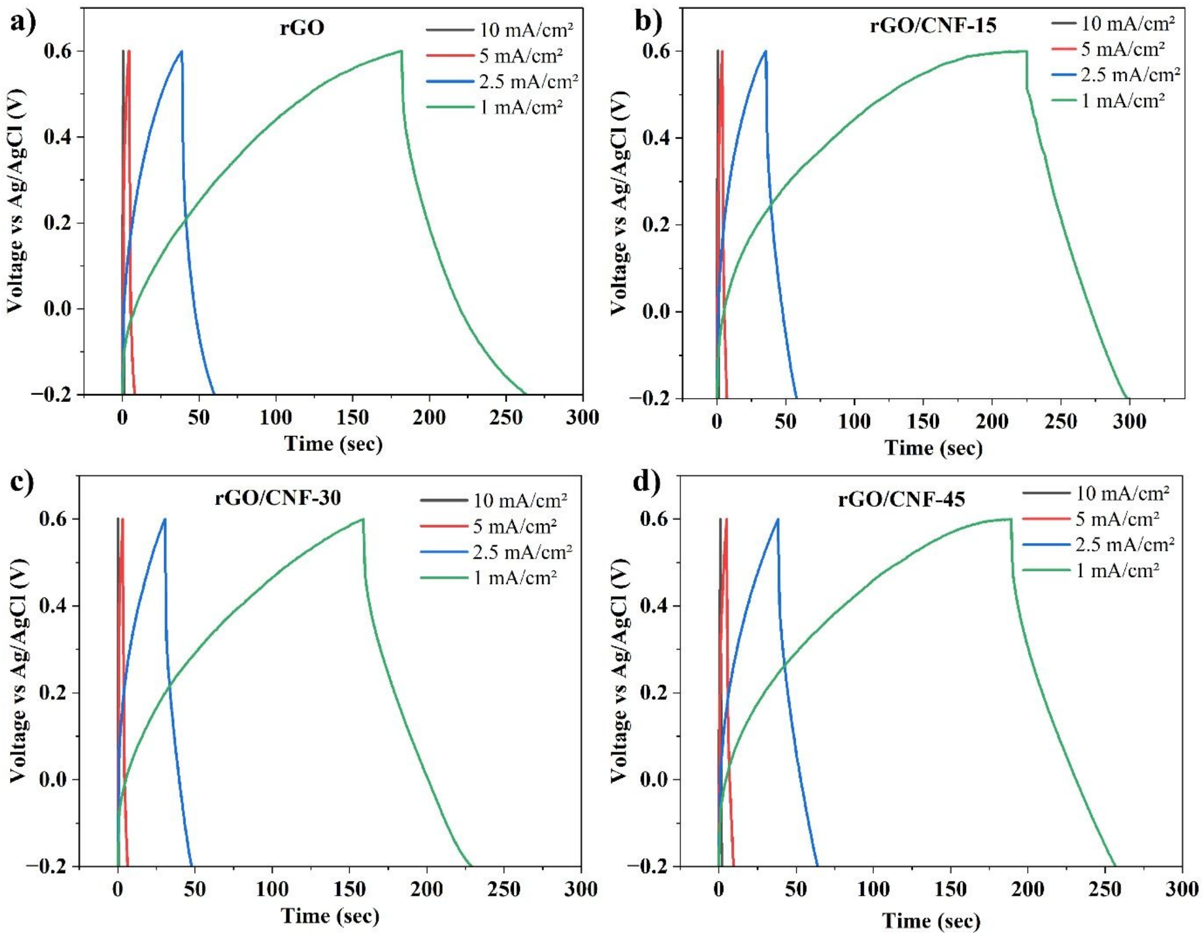


**Figure S3.** GCD curves of a) rGO, b) rGO/CNF-15, c) rGO/CNF-30, and d) rGO/CNF-45 film electrodes at different current densities from 1 to 10 mA/cm$^2$.
